# Intracellular free radical production by peripheral blood T lymphocytes from patients with systemic sclerosis: role of NADPH oxidase and ERK1/2

**DOI:** 10.1186/s13075-015-0591-8

**Published:** 2015-03-17

**Authors:** Donatella Amico, Tatiana Spadoni, Marina Rovinelli, Marta Serafini, Giovanna D’Amico, Nadia Campelli, Silvia Svegliati Baroni, Armando Gabrielli

**Affiliations:** Dipartimento Scienze Cliniche e Molecolari, Università Politecnica delle Marche, Via Tronto 10, 60020 Ancona, Italy; Centro Ricerca M. Tettamanti, Clinica Pediatrica Università Milano-Bicocca, Ospedale San Gerardo, Via Donizetti 106, 20900 Monza, Italy

## Abstract

**Introduction:**

Abnormal oxidative stress has been described in systemic sclerosis (SSc) and previous works from our laboratory demonstrated an increased generation of reactive oxygen species (ROS) by SSc fibroblasts and monocytes. This study investigated the ability of SSc T lymphocytes to produce ROS, the molecular pathway involved, and the biological effects of ROS on SSc phenotype.

**Methods:**

Peripheral blood T lymphocytes were isolated from serum of healthy controls or SSc patients by negative selection with magnetic beads and activated either with PMA or with magnetic beads coated with anti-CD3 and anti-CD28 antibodies. Intracellular ROS generation was measured using a DCFH-DA assay in a plate reader fluorimeter or by FACS analysis. CD69 expression and cytokine production were analyzed by FACS analysis. Protein expression was studied using immunoblotting techniques and mRNA levels were quantified by real-time PCR. Cell proliferation was carried out using a BrdU incorporation assay.

**Results:**

Peripheral blood T lymphocytes from SSc patients showed an increased ROS production compared to T cells from healthy subjects. Since NADPH oxidase complex is involved in oxidative stress in SSc and we found high levels of gp91phox in SSc T cells, SSc T cells were incubated with chemical inhibititors or specific siRNAs against gp91phox. Inhibition of NADPH oxidase partially reverted CD69 activation and proliferation rate increase, and significantly influenced cytokine production and ERK1/2 activation.

**Conclusions:**

SSc T lymphocityes are characterized by high levels of ROS, generated by NADPH oxidase via ERK1/2 phosphorylation, that are essential for cell activation, proliferation, and cytokine production. These data confirm lymphocytes as key cellular players in the pathogenesis of systemic sclerosis and suggest a crucial link between ROS and T cell activation.

**Electronic supplementary material:**

The online version of this article (doi:10.1186/s13075-015-0591-8) contains supplementary material, which is available to authorized users.

## Introduction

Systemic sclerosis or scleroderma (SSc) is a connective tissue disease of unknown origin characterized by excessive deposition of collagen and other extracellular matrix components in skin and visceral organs, severe alterations in the microvasculature, and humoral and cellular immunologic abnormalities [1,2].

Although the pathogenesis of SSc is unclear, several features suggest that activation of immune cells plays a central role in the development of the disease [3-6].

Upregulation of collagen synthesis in fibroblasts adjacent to infiltrating T cells suggests that T cells can trigger fibroblast activation both by direct contact and by paracrine action through the production of soluble mediators [7-10]. On the other hand, chemokines secreted by activated fibroblasts can induce chemotaxis of inflammatory cells, contributing to the amplification of the pathogenetic process [11].

Reactive oxygen species (ROS) have long been considered deleterious byproducts of mitochondrial and endosomal metabolic activities. Over the past few years, numerous studies have demonstrated that T lymphocytes also produce ROS, upon T cell receptor (TCR) stimulation [12] or after treatment with lectins (concanavalin A (conA) or phytohemagglutinin (PHA) [13-15] and mitogens (phorbol myristate acetate (PMA) or superantigens) [13,16,17]. ROS production in T cells is involved in the regulation of T helper (Th)1/Th2 balance, in T cell maturation, proliferation and survival through the modulation of signal transduction [18]. The available data on the mechanism used by T cells to produce ROS are limited, although lipid metabolism, mitochondria and NADPH oxidase seem to be the most important sources [19,20].

Following Murrell’s hypothesis [21,22], several reports have provided indirect [23,24] and direct evidence [25] of abnormal ROS generation in SSc. We have previously demonstrated that monocytes and fibroblasts from SSc patients represent a source of free radical species [25,26]. In this study, we present data demonstrating that unstimulated T lymphocytes from SSc patients are able to produce ROS, and we show the molecular pathways involved in ROS generation.

## Methods

### Human subjects

Thirty-four nonsmoking SSc patients (six men and twenty-eight women) with a median age of 57 years (range 27 to 84) were studied. The clinical features of SSc population are presented in Table S1 in Additional file 1. Diagnosis was made following the American College of Rheumatology preliminary criteria for the classification of SSc [27], and the patients were classified into the diffuse SSc and limited SSc subset according to LeRoy *et al*. [28]. The study was approved by the Institutional Ethics Committee of Università Politecnica delle Marche, Ancona, Italy. An informed written consent was obtained from all patients. At the time of the investigation the patients, who had never been on immunosuppressive therapy, had not received any treatment for the previous six weeks. Seventeen age-, sex- and race-matched, normal, nonsmoking, healthy volunteers were also evaluated and constituted the control population.

### Isolation of lymphocytes

Mononuclear cell suspensions were prepared from heparin-collected peripheral blood (PB) of patients and healthy controls by Ficoll-Hypaque density gradient centrifugation. Peripheral blood lymphocytes (PBL) were separated after removal of monocytes by adherence. In selected experiments residual monocytes and B lymphocytes were further depleted by incubating with CD14 and CD19 magnetic microbeads according to the manufacturer’s instructions (Miltenyi Biotec, Bergisch Gladbach, Germany). Otherwise, PB T cells were purified using a negative isolation procedure (T Cell Negative Isolation kit; Dynal; Invitrogen, Carlsbad, CA, USA), which resulted in a 95% CD3+ cell population, as assessed by FACS analysis after staining with a peridinin chlorophyl protein (PerCP)-conjugated anti-CD3 monoclonal antibody (Becton Dickinson, Franklin Lakes, NJ, USA). T cells were then resuspended at 1 × 10^6^ cells/ml in RPMI 1640 medium, supplemented with 10% fetal calf serum (FCS), 2 mM L-glutamine, 100 U/ml penicillin/streptomycin, at 37°C in a humidified atmosphere with 5% CO_2_.

### ROS determination

Relative changes in intracellular ROS were monitored using the fluorescent probe dichloro-dihydro-fluorescein diacetate (DCFH-DA) (Life Technologies, Carlsbad, CA, USA). Cells were incubated with different inhibitors, PD98059 (50 μM, 2 hours) (Calbiochem, Billerica, MA, USA), diphenyleneiodonium (DPI, 20 μM, 1 hour) (Calbiochem), N-acetyl-cysteine (NAC 10 mM, 1 hour) (Sigma-Aldrich, St Louis, MO, USA), or cytokines, interleukin (IL)-1 (10 ng/ml), IL-4 (15 ng/ml), IL-6 (5 ng/ml), IL-13 (50 ng/ml), interferon alpha (IFNα) (0.5 ng/ml), tumor necrosis factor alpha (TNFα) (1 ng/ml), transforming growth factor beta (TGFβ) (1 ng/ml), and platelet-derived growth factor beta (PDGF-BB) (15 ng/ml) for 15 minutes. Cells were then stained with 20 μM DCFH-DA for 20 minutes and analyzed in a plate reader fluorimeter (Wallac, Becton Dickinson). ROS production was also detected by FACS analysis. In brief, cells were treated and stained with 2 μM DCFH-DA for 30 minutes and fluorescence was measured using an argon ion laser at 488 nm excitation and 510 to 540 nm emission on a FACscan flow cytometer (Becton Dickinson).

### Western blots

Total cell lysates were separated by sodium dodecyl sulfate (SDS)-polyacrylamide gels as already described [26]. Antibodies against ERK2, pERK1/2, Ha-Ras, and gp91phox (Santa Cruz Biotechnology, Dallas, TX, USA) were used.

### Immunofluorescence

For double staining, PBL were incubated simultaneously with 2 μM DCFH-DA and PerCP-conjugated anti-CD3 antibody (Becton Dickinson) for 20 minutes and analyzed on a FACscan flow cytometer.

### Small interference RNA transfection

T lymphocytes were transfected with 100 nM small interfering RNA (siRNA) specific for gp91phox (Dharmachon, Lafayette, CO, USA) or control siRNA using Lipofectamine™ 2000 reagent, following the manufacturer’s instructions (Life Technology). After 4 hours, 10% FCS and 20 U/ml IL-2 were added to the culture medium. Gene silencing and ROS production were monitored 72 hours after transfection.

### Real-time PCR (RT-PCR)

Total RNA was isolated with Pure Link RNA Minikit (Life Technologies) and reverse transcribed using IScript cDNA Synthesis Kit (Bio-Rad Laboratories, Hercules, CA, USA) according to the manufacturer’s instructions. Gene expression was quantified by SYBR Green real-time PCR in a iCycler iQ™ real-time PCR Detection System (Bio-Rad Laboratories). Specific primer pairs for each gene were designed with the Universal ProbeLibrary Assay Design Center by Roche Applied Science (Penzberg, Germany) and were as follows: gp91phox 5′-TCACTTCCTCCACCAAAACC-3′ (forward), 5′GGGAT-TGGGCATTCCTTTAT3′ (reverse); GAPDH 5′-TGCACCACCAACTGCTTAGC-3′ (forward), 5′-TGGGATTTCCATTGATGACAAGC-3′ (reverse). The relative expression was calculated using the 2^_ΔΔ^Ct formula.

### Proliferation assay

T cells were treated with DPI (10 μM) and PD98059 (20 μM) for 48 hours and then pulsed with bromodeoxyuridine (BrdU) for 6 hours. Proliferation was measured with a colorimetric immunoassay (Roche Diagnostics, Mannheim, Germany).

### Viability assay

After specific treatments, cells were incubated with 20 mg/ml 7-aminoactinomycin D (7-AAD) (Sigma-Aldrich) for 20 minutes at 4°C in the dark and then acquired on a FACScan flow cytometer.

### Cytokine determination

For intracellular staining assay, isolated T cells were treated with 20 μM DPI for 1 hour and then stimulated with 100 ng/ml PMA and 20 U/ml IL-2 for 4 hours. After 2-hour incubation with Brefeldin A (Sigma-Aldrich), cells were permeabilized with Cyto Fix/Perm (Becton Dickinson). Intracellular staining was performed with PerCP-conjugated anti-IL-4 antibody (BioLegend, San Diego, CA, USA) or fluorescein isothiocyanate (FITC)-conjugated anti-interferon γ (IFNγ) antibody (Becton Dickinson). Cells were then analyzed by FACS analysis.

### Statistical analysis

Data are expressed as means ± standard deviation (SD). Mean values were compared using Student’s paired and unpaired *t* test. *P* values less than 0.05 were considered significant.

## Results

### Spontaneous ROS production by peripheral blood lymphocytes from SSc patients

**Figure 1:**
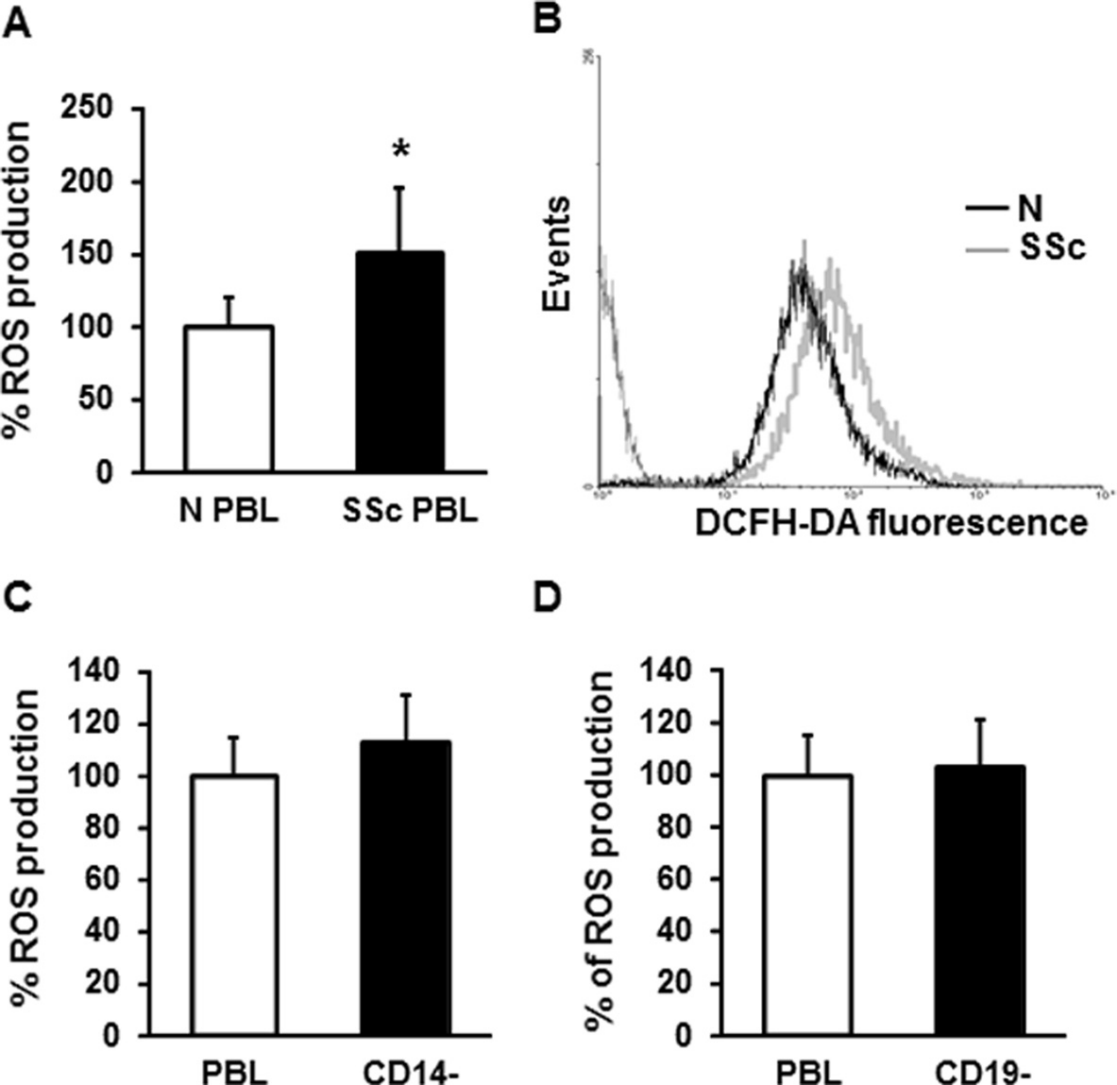
**ROS production by PBL from SSc patients and healthy controls. (A)** PBL from 17 healthy controls (white bar) and 34 SSc patients (black bar), isolated from peripheral blood were stained with 20 μM DCFH-DA for 20 minutes, and fluorescence was measured in a plate reader fluorimeter. Each patient and control was tested three times and the mean value used to calculate the mean of each group. Data are means ± standard deviation (SD). ^*^ *P* <0.05 compared to normal PBL. **(B)** ROS production by PBL from one healthy control (black line) and one SSc patient (grey line) was analyzed by FACS analysis. A representative histogram of three independent experiments is shown. **(C and D)** SSc CD14- (C) and SSc CD19- cells (D) were purified from PBL using CD14 and CD19 microbeads. The total fractions of cells (PBL, white bars) and the collected depleted fractions (black bars) were stained with 20 μM DCFH-DA for 20 minutes, and fluorescence was measured on a plate reader fluorimeter. Data are means ± SD of three independent experiments with cells from three distinct subjects. DCFH-DA, 2′, 7′-dichlorodihydrofluorescin diacetate; PBL, peripheral blood lymphocytes; ROS, reactive oxygen species; SSc, systemic sclerosis.

**Figure 2:**
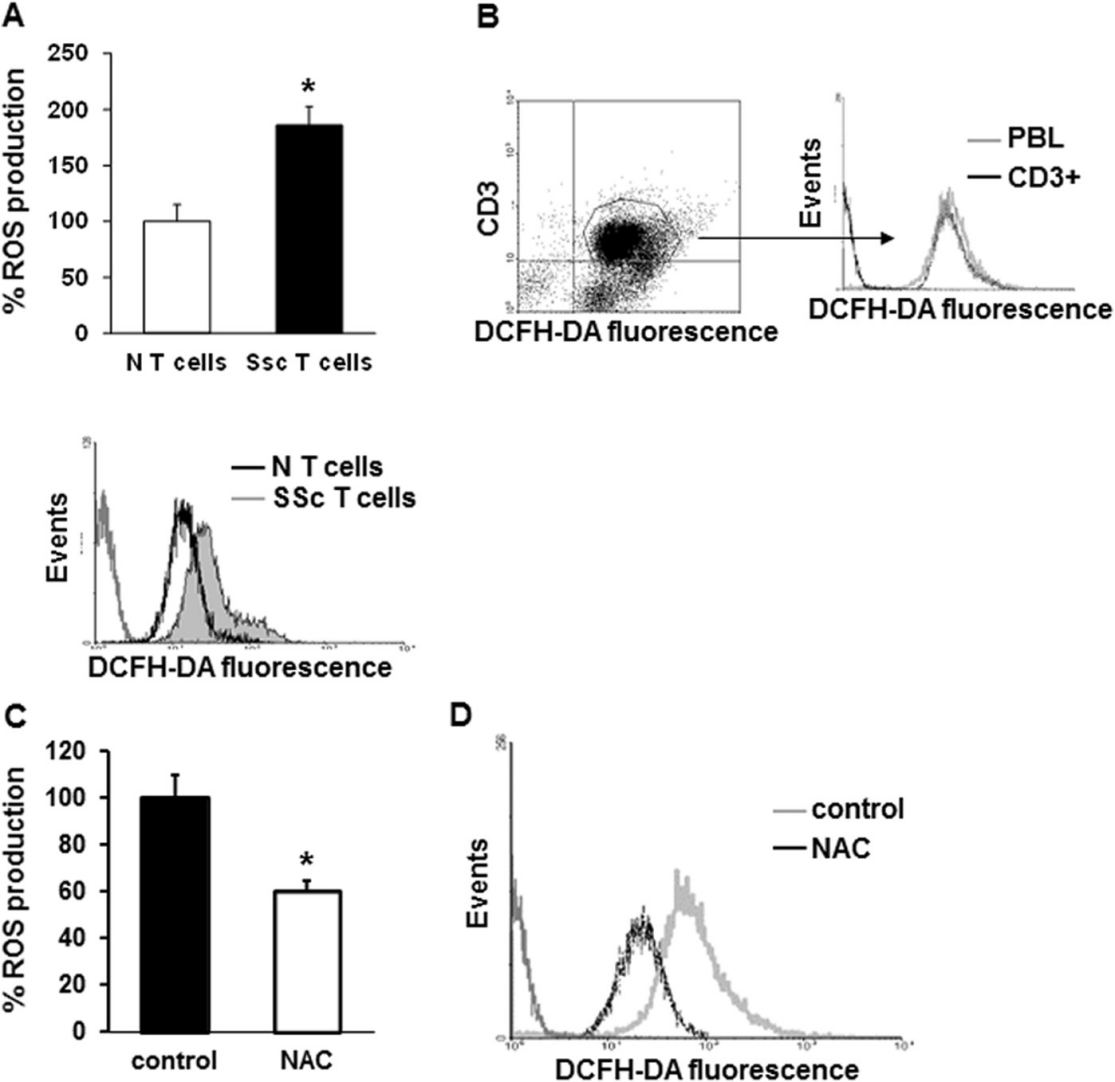
**ROS production by T cells from SSc patients and healthy controls. (A)** T lymphocytes from 12 healthy controls (white bar) and 23 SSc patients (black bar) purified using a negative selection procedure were stained with 20 μM DCFH-DA, and the fluorescence was quantified in a plate reader fluorimeter (upper panel). Each patient and control was tested three times and the mean value used to calculate the mean of each group. Data are means ± standard deviation (SD). ^*^ *P* <0.05 compared to normal T cells. Representative histogram of ROS production in T cells from one healthy control (black line) and one SSc patient (grey line) analyzed by FACS analysis is shown in the lower panel. **(B)** ROS production in total (grey line) and in CD3+ PBL (black line) from one SSc patient simultaneously stained with 2 μM DCFH-DA and monoclonal PerCP-conjugated anti-CD3 antibody for 20 minutes was analyzed by FACS analysis. Histogram is representative of three independent experiments. **(C)** CD3+ T lymphocytes isolated from 10 SSc patients and treated with NAC (10 mM, 1 hour) were stained with 20 μM DCFH-DA for 20 minutes, and the fluorescence was measured in a plate reader fluorimeter. Each treatment was tested three times and the mean value used to calculate the mean of each group. Data are means ± SD. ^*^ *P* <0.05 compared to untreated T cells (control). **(D)** Untreated (control, grey line) or treated with NAC CD3+ T cells (black line) were simultaneously stained with 2 μM DCFH-DA and monoclonal PerCP-conjugated anti-CD3 antibody for 20 minutes, and ROS production was analyzed by FACS analysis. Representative histogram of three independent experiments is shown. DCFH-DA, 2′, 7′-dichlorodihydrofluorescin diacetate; NAC, N-acetylcysteine; PBL, peripheral blood lymphocytes; PerCP, peridinin chlorophyl protein; ROS, reactive oxygen species; SSc, systemic sclerosis.

We previously demonstrated that unstimulated monocytes isolated from SSc patients released large amount of reactive oxygen species (ROS) [25]. These data led us to investigate whether other blood cell types were involved in the oxidative burst that characterizes SSc. Peripheral blood lymphocytes (PBL) were obtained from 17 healthy individuals and 34 SSc patients (Table S1 in Additional file 1) and analyzed for ROS production. PBL from SSc patients produced a significantly larger amount of ROS compared to healthy controls as measured in a plate reader fluorimeter (150 ± 45 and 100 ± 20 respectively, Figure 1A) and by FACS analysis (100 and 46.2 ± 8 respectively, Figure 1B) (*P* <0.05). We then analyzed the relationship between the amount of ROS generated by PBL and the clinical features of SSc patients. No difference was detected when all patients were divided into the limited or the diffuse subset (17 limited SSc patients 132.8 ± 56 vs. 17 diffuse SSc patients 163.3 ± 48, *P* = 0.066), or into early (less than 5 years) or late (6 years or more) disease (17 early SSc patients 157.37 vs. 17 late SSc patients 161.25, *P* = 0.45). In order to identify the PB cell subpopulation responsible for the increased ROS production in SSc samples, we purified cell populations depleted of CD14 (CD14-) and CD19 (CD19-) fractions by magnetic beads. Since no difference in ROS generation between PBL and the purified cell populations was observed, we could assume that neither CD14+ nor CD19+ cells were responsible for the oxidative burst in SSc samples (Figure 1C and D respectively). To test whether ROS generation by PBL was due to T cells, we performed a negative selection procedure specific for T cell isolation in 12 controls and in 23 SSc samples and ROS production was analyzed. Figure 2A shows that T cells from SSc patients, even in absence of deliberate stimulation, produced significantly larger amount of ROS compared to T cells from healthy controls (186 ± 17 and 100 ± 15 respectively, *P* <0.05). Data were confirmed by FACS analysis (Figure 2A, lower panel). No difference was detected when all patients were divided into the limited or the diffuse subset (*P* = 0.13). The simultaneous staining of SSc PBL with DCFH-DA and anti-CD3 PerCP-conjugated antibody confirmed the implication of T cell population in ROS production. To understand which subpopulation of T cells was implicated in ROS production, we stained SSc PBL with DCFH-DA and PE-conjugated anti-CD4 (Figure S1A in Additional file 2) or anti-CD8 antibody (Figure S1B in Additional file 2). We observed that both CD4+ and CD8+ lymphocytes were involved in ROS generation. To further validate the involvement of CD3+ T cells in ROS generation, CD3+ T cells from SSc patients were treated with 10 mM N-acetylcysteine (NAC), a ROS scavenging agent, and a significant reduction in free radical production was observed (100 ± 10 and 60 ± 5 respectively, *P* <0.05) (Figure 2C). These results were confirmed by FACS analysis (Figure 2D).

### Molecular pathway involved in ROS production in SSc T lymphocytes

**Figure 3:**
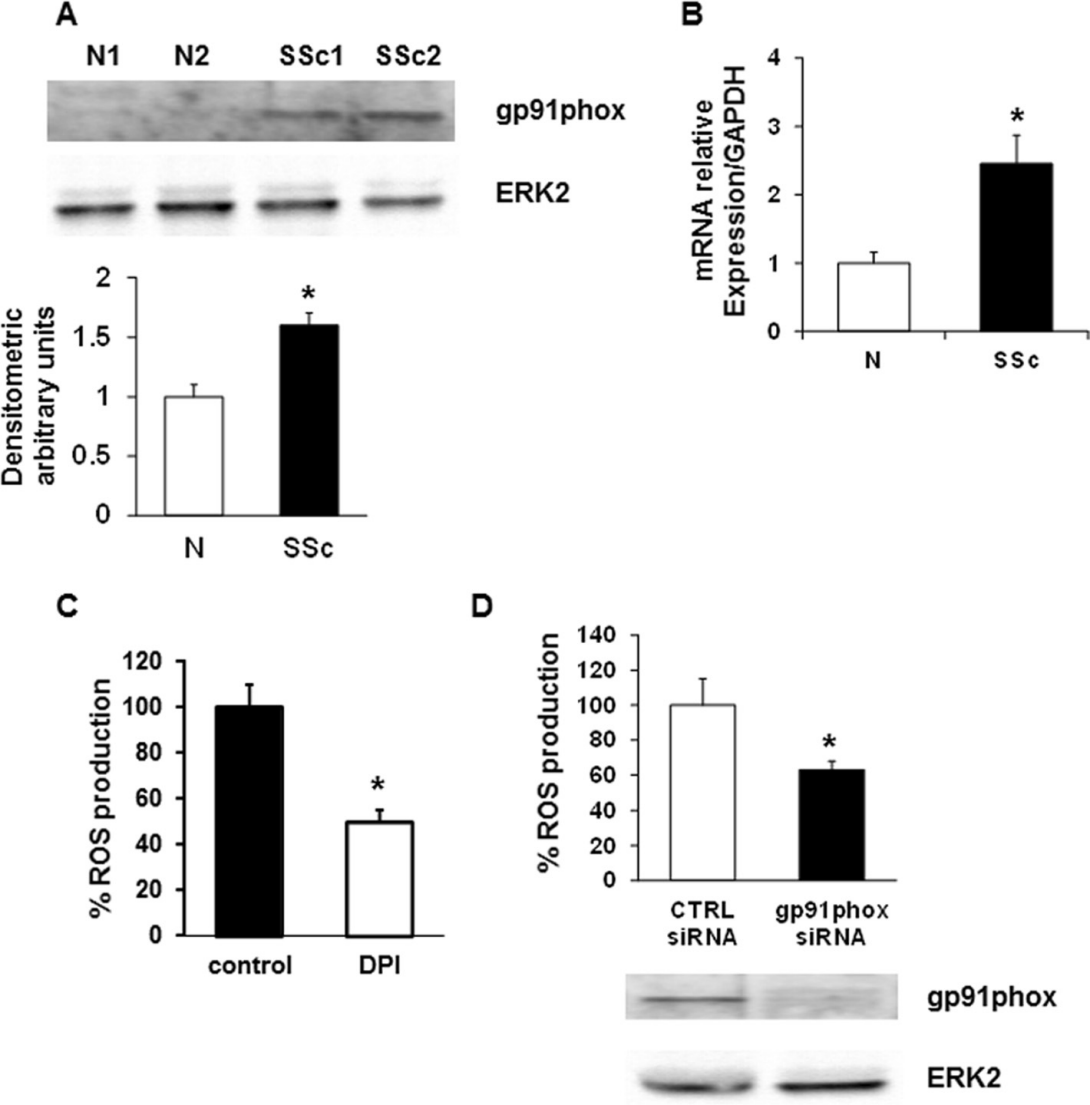
**Role of NOX2 in ROS production by SSc T cells. (A)** A representative gp91phox immunoblot of three independent experiments is shown. T cells were isolated from healthy controls (N) and SSc patients (SSc) and then analyzed by immunoblotting with specific antibodies. The lower panel shows the densitometric analysis from distinct experiments with five healthy controls and five SSc patients. Data are means ± standard deviation (SD). ^*^ *P* <0.05 compared to normal T cells. **(B)** Real time-PCR for gp91phox expression in T cells was isolated from five healthy controls and five SSc patients. Each patient and control was tested three times and the mean value used to calculate the mean of each group. Data are means ± SD. ^*^ *P* <0.05 compared to normal T cells. **(C)** T cells isolated from five SSc patients were treated with DPI (20 μM, 1 hour), stained with 20 μM DCFH-DA and subsequently analyzed in a plate reader fluorimeter. Each treatment was tested three times and the mean value used to calculate the mean of each group. Data are means ± SD. ^*^ *P* <0.05 compared to untreated T cells (control). **(D)** ROS production in SSc T lymphocytes transiently transfected with control siRNA (CTRL siRNA) or gp91phox targeted siRNA (gp91phox siRNA) for 72 hours (upper panel). Data are means ± SD of three independent experiments with cells from three distinct patients. ^*^ *P* <0.05 compared to control siRNA. Transfection efficiency was monitored by western blot (lower panel). DCFH-DA, 2′, 7′-dichlorodihydrofluorescin diacetate; NOX2, NADPH oxidase 2; ROS, reactive oxygen species; siRNA, small interfering RNA; SSc, systemic sclerosis.

Since human T cells express a functional gp91phox (NOX2) [29], we were interested to investigate whether this enzymatic complex was also implicated in ROS production in SSc T lymphocytes. SSc T cells expressed higher amount of gp91phox at protein (Figure 3A) and transcriptional levels (Figure 3B) compared to healthy controls. Treatment of SSc T cells with DPI, a specific inhibitor of flavonoid-containing enzymes such as NADPH oxidase, determined a 50% reduction in ROS production (*P* <0.05), confirming the important role of NADPH oxidase in ROS generation (Figure 3C). Moreover, transfection of SSc T cells with siRNA specific for gp91phox led to a significant decrease of ROS generation compared to cells transfected with a control siRNA (100 ± 15 and 63 ± 5 respectively, *P* <0.05) (Figure 3D).

**Figure 4:**
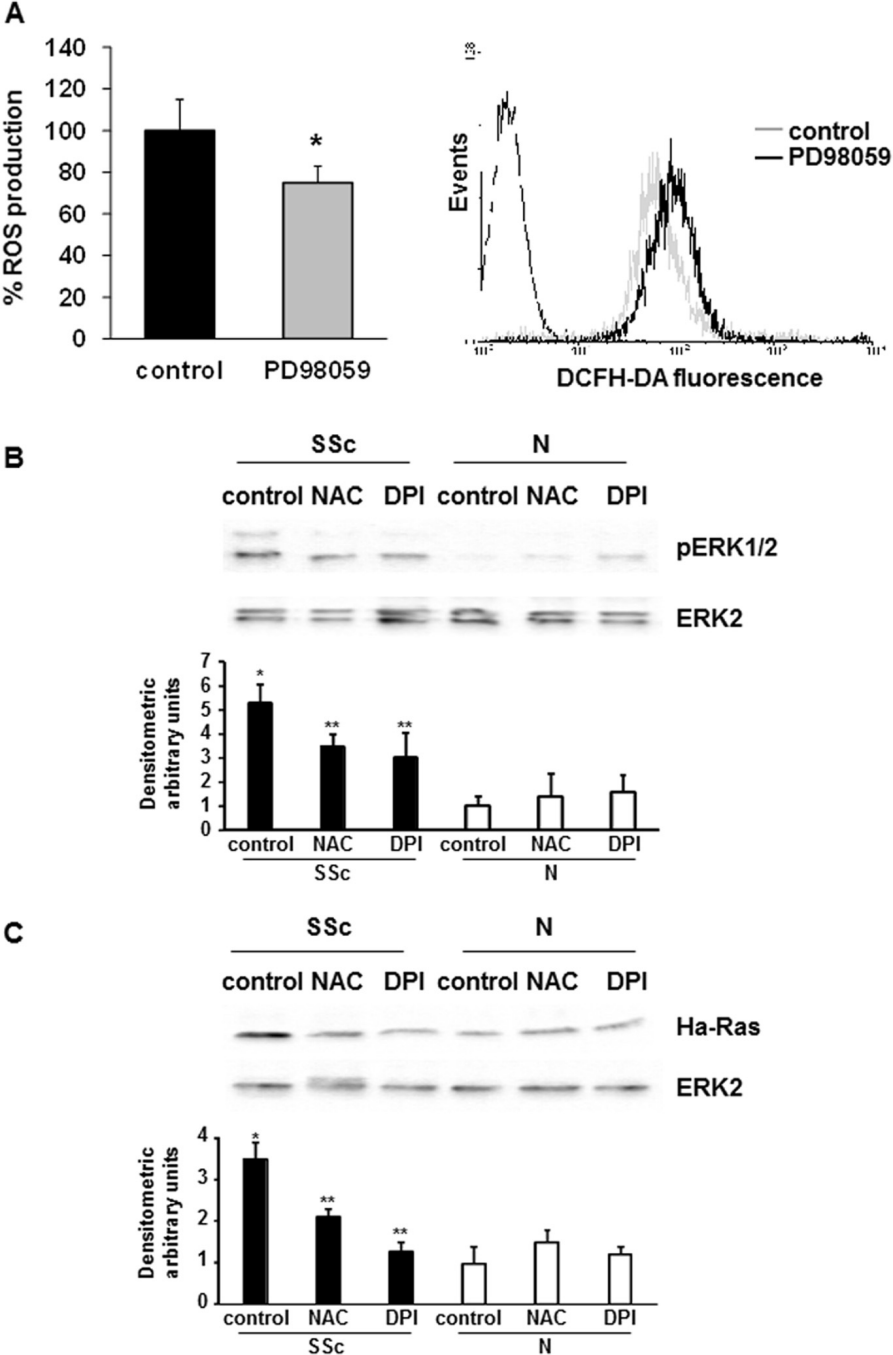
**pERK1/2 implication in ROS production. (A)** T lymphocytes from five SSc patients were incubated with PD98059 (50 μM, 2 hours) or left untreated. After staining with 20 μM DCFH-DA, fluorescence was analyzed in a plate reader fluorimeter (left panel). Each treatment was tested three times and the mean value used to calculate the mean of each group. Data are means ± standard deviation (SD). ^*^ *P* <0.05 compared to untreated T cells (control). Representative histogram of ROS production by SSc T cells is shown in the right panel. Untreated (control, grey line) and PD98059 treated cells (black line) were stained with 2 μM DCFH-DA and analyzed by FACS analysis. **(B)** Representative pERK1/2 immunoblot of three independent experiments is shown (upper panel). T lymphocytes were isolated from five healthy controls and five SSc patients, treated with NAC (10 mM, 1 hour) or DPI (20 μM, 1 hour), and then analyzed by immunoblotting with specific antibodies. The lower panel shows the densitometric analysis from distinct experiments with five healthy controls and five SSc patients. Data are means ± SD. ^*^ *P* <0.05 compared to untreated normal T cells. ^**^ *P* < 0.05 compared to untreated SSc T cells. **(C)** Representative Ha-Ras immunoblot of three independent experiments is shown (upper panel). T lymphocytes were isolated from five healthy controls and five SSc patients, treated with NAC (10 mM, 1 hour) or DPI (20 μM, 1 hour), and then analyzed by immunoblotting with specific antibodies. The lower panel shows the densitometric analysis from distinct experiments with five healthy controls and five SSc patients. Data are means ± SD. ^*^ *P* <0.05 compared to untreated normal T cells. ^**^ *P* <0.05 compared to untreated SSc T cells. DCFH-DA, 2′, 7′-dichlorodihydrofluorescin diacetate; DPI, diphenylene iodonium; NAC, N-acetylcysteine; ROS, reactive oxygen species; SSc, systemic sclerosis.

The implication of extracellular signal-regulated kinase (ERK)1/2 signaling in ROS production has been assessed in different cell types, including T lymphocytes [18], and in a previous work, we described an intracellular loop that involves Ha-Ras, ERK1/2, and ROS, leading to increased collagen gene expression in SSc fibroblasts [30]. To evaluate the involvement of ERK1/2 signaling in ROS production in SSc T cells, we treated T lymphocytes from SSc patients with PD98059, an ERK1/2 inhibitor. As shown in Figure 4A, PD98059 significantly reduced DCFH-DA fluorescence intensity compared to untreated cells (100 ± 15 and 75 ± 8, *P* <0.05), confirming ERK1/2 implication in ROS production in SSc T cells.

It has been reported that ROS produced by the NADPH oxidase system could modulate MEK/ERK pathway activation [12]. In order to validate this hypothesis, we examined the activation levels of ERK1/2 in SSc T lymphocytes in the presence or absence of NAC or DPI. Figure 4B shows that in basal conditions, SSc samples expressed higher levels of pERK1/2 compared to healthy controls. Inhibition of NADPH oxidase with DPI or treatment with NAC in SSc T cells determined a reduction of activated ERK1/2 levels, demonstrating an important role for ROS in the upstream activation signaling of MEK/ERK in SSc T cells.

We then compared Ha-Ras levels in SSc and normal T lymphocytes and the modulation of Ha-Ras by ROS production. We observed that SSc T lymphocytes showed higher levels of Ha-Ras compared to control cells. Moreover, treatment with NAC or DPI led to a significant decrease in Ha-Ras expression, confirming the activated state of SSc lymphocytes due to oxidative stress (Figure 4C).

### Modulation of ROS production by cytokines

**Figure 5:**
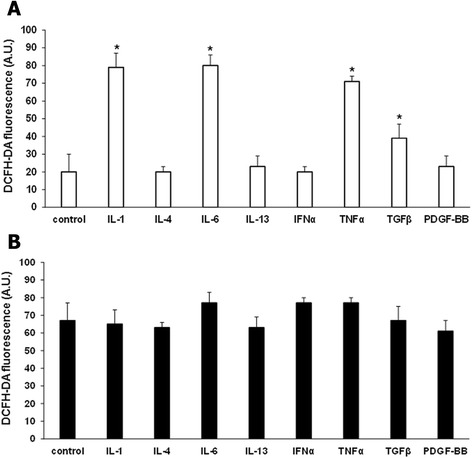
**Modulation of ROS production by cytokines.** ROS production by T cells isolated from three healthy controls **(A)** and three SSc patients **(B)** and treated with IL-1 (10 ng/ml), IL-4 (15 ng/ml), IL-6 (5 ng/ml), IL-13 (50 ng/ml), IFNα (0.5 ng/ml), TNFα (1 ng/ml), TGFβ (1 ng/ml), and PDGF-BB (15 ng/ml) for 15 minutes. Cells were then stained with 20 μM DCFH-DA and analyzed in a plate reader fluorimeter. Data are means ± standard deviation (SD) of three independent experiments with cells from three distinct subjects. ^*^ *P* <0.05 compared to untreated T cells. DCFH-DA, 2′, 7′-dichlorodihydrofluorescin diacetate; IFNα, interferon alpha; IL, interleukin; PDGF-BB, platelet-derived growth factor beta; ROS, reactive oxygen species; SSc, systemic sclerosis; Th, T helper; TGFβ, transforming growth factor beta; TNFα, tumor necrosis factor alpha.

To verify whether soluble mediators were involved in ROS generation in T lymphocytes, we treated SSc and normal T cells with IL-1 (10 ng/ml), IL-4 (15 ng/ml), IL-6 (5 ng/ml), IL-13 (50 ng/ml), IFNα (0.5 ng/ml), TNFα (1 ng/ml), TGFβ (1 ng/ml), and PDGF-BB (15 ng/ml) for 15 minutes before ROS analysis. IL-1, IL-6, TNFα, and TGFβ induced a significant increase in ROS generation in normal T cells (78 ± 5, 77 ± 7, 70 ± 5, 38 ± 5 respectively compared to untreated cells 18 ± 10, *P* <0.05) (Figure 5A). In SSc T cells instead ROS levels were not modified by incubation with the same cytokines and growth factors (Figure 5B), suggesting that SSc T cells were already maximally activated and were not responsive to external stimuli.

### Biological effects of ROS in activated T cells

Under experimental conditions, functional analysis of T lymphocytes requires *in vitro* stimulation that mimics as closely as possible physiological *in vivo* activation of T cells [31]. We therefore incubated normal and SSc T lymphocytes with PMA and tested ROS production and ERK1/2 phosphorylation. Both normal and SSc cells showed overproduction of intracellular ROS (Figure S2A in Additional file 3) and upregulation of pERK levels after PMA incubation (Figure S2B in Additional file 3).

**Figure 6:**
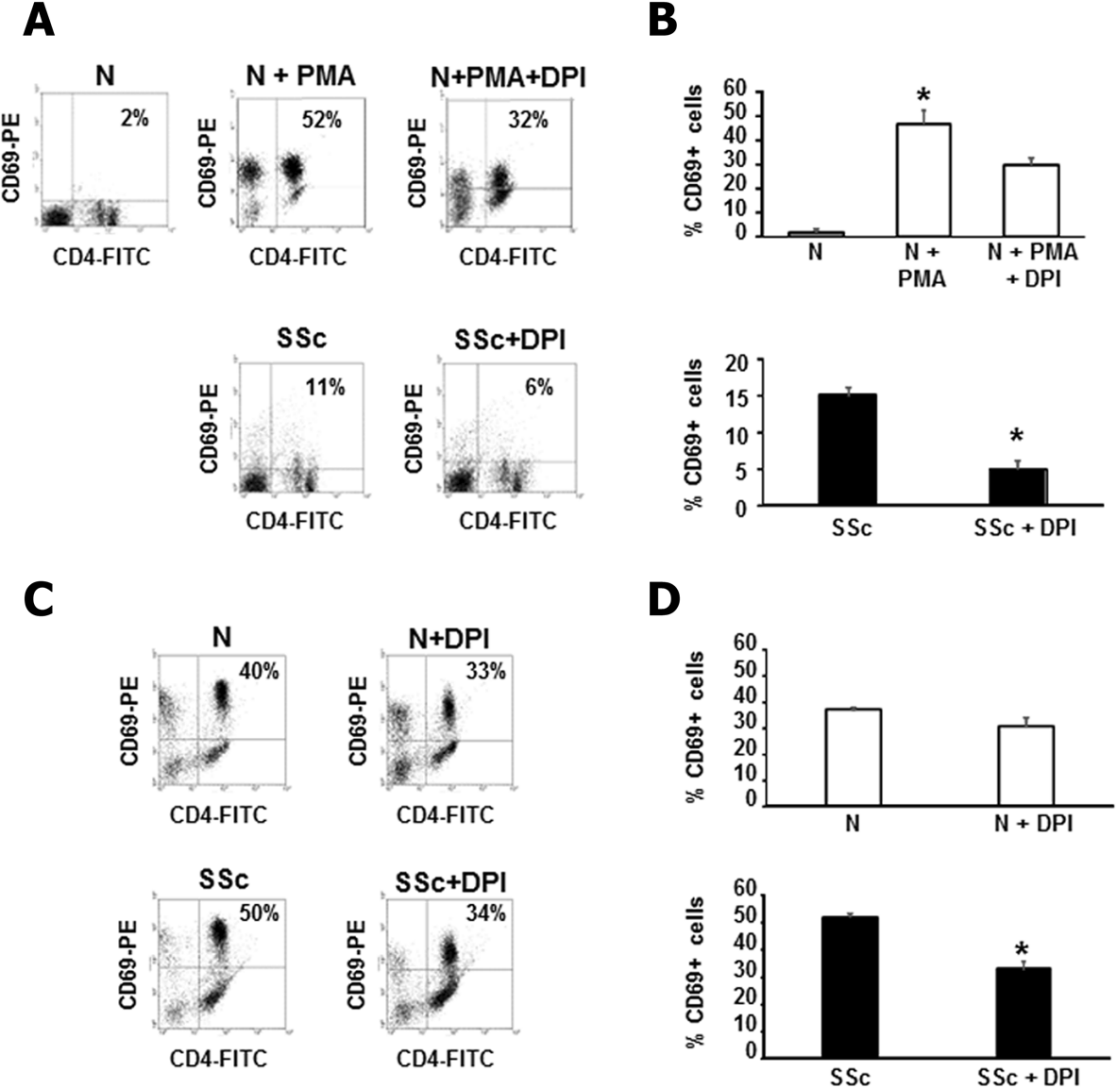
**Modulation of T cell activation by ROS. (A)** One representative flow cytometry analysis of CD69 expression is shown. PMA activated normal T cells (upper panels) or SSc T cells (lower panels) were treated with DPI (20 μM, 1 hour). Plots represent live cells gated for CD4+ cells. **(B)** Percentage of CD69+ cells in three healthy controls (upper panel) and three SSc patients (lower panel) are presented. Cells were treated as in A. Data are means ± standard deviation (SD). ^*^ *P* <0.05 compared to untreated T cells. **(C)** One representative flow cytometry analysis of CD69 expression is shown. Normal T cells (upper panels) or SSc T cells (lower panels) were activated with CD3/CD28 magnetic beads and treated with DPI (20 μM, 1 hour). Plots represent live cells gated for CD4+ cells. **(D)** Percentage of CD69+ cells in three healthy controls (upper panel) and three SSc patients (lower panel) are presented. Cells were treated as in C. Data are means ± SD. ^*^ *P* <0.05 compared to untreated T cells. DPI, diphenylene iodonium; PMA, phorbol myristate acetate; ROS, reactive oxygen species; SSc, systemic sclerosis.

PMA activation of CD4+ T cells was then assessed by monitoring the expression of CD69, an early activation marker on the surface of antigen-specific activated lymphocytes *in vitro* [32]. As shown in the upper panels of Figure 6A and B, incubation of normal T cells with PMA led to an increase of the activation rate on CD4+ cells (47% increase compared to basal, *P* <0.05), that was partially reverted by treatment with DPI (30% increase compared to basal). On the other hand, CD69 was upregulated in SSc T cells compared to normal controls even in basal conditions (15% increase compared to normal cells, Figure 6B), and NADPH oxidase inhibition reverted the activated state of SSc T lymphocytes (lower panels of Figure 6A and B).

To provide a more physiological mechanism of T cell activation, we stimulated lymphocytes with magnetic beads coated with anti-CD3 and anti-CD28 antibodies, mimicking the binding of antigen-presenting cells to T cells [33,34]. CD3-CD28 beads stimulation induced an increase of ROS levels (Figure S3A in Additional file 4) and CD69 expression in SSc and normal T cells (Figure 6C). Treatment with DPI downregulated ROS production (Figure S3B in Additional file 4) as well as the levels of the activation marker (Figure 6C and D).

**Figure 7:**
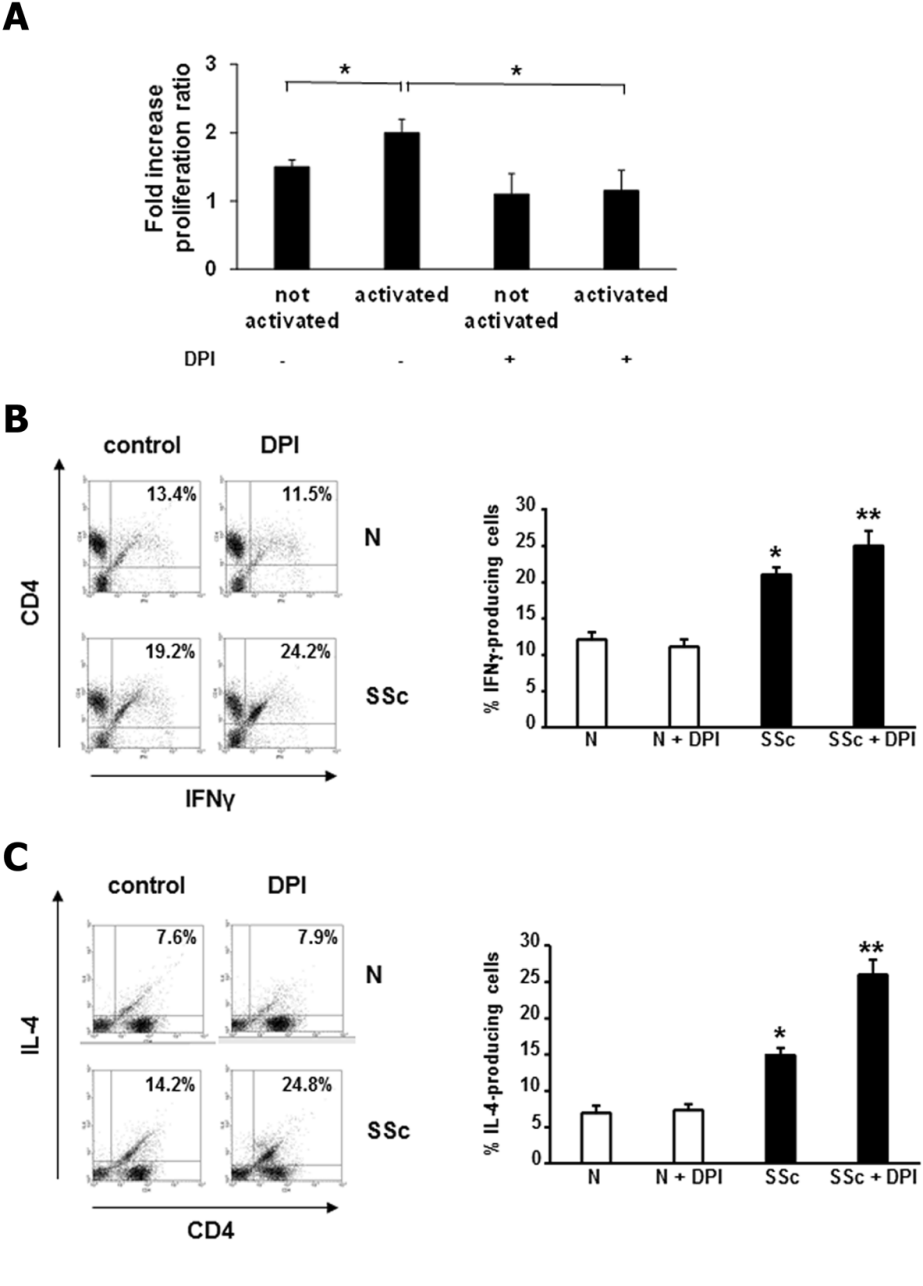
**Modulation of proliferation and cytokine production by ROS in activated T cells. (A)** T cells from three healthy controls and three SSc patients were activated with CD3/CD28 magnetic beads, treated with DPI (20 μM, 1 hour) and pulsed with BrdU for 6 hours. Proliferation was measured with a colorimetric immunoassay in a plate reader fluorimeter. Data are means ± standard deviation (SD) of fold increase of SSc/N proliferation ratio. ^*^ *P* <0.05. **(B)** One representative flow cytometry analysis of IFNγ-producing T cells is shown. Normal T cells (upper panels) or SSc T cells (lower panels) were activated with CD3/CD28 magnetic beads and treated with DPI (20 μM, 1 hour). Plots represent live cells gated for CD4+ cells. Percentage of IFNγ-producing T cells in three healthy controls and three SSc patients are presented (right panel). Data are means ± SD. ^*^ *P* <0.05 compared to normal untreated T cells. ^**^ *P* <0.05 compared to SSc untreated T cells. **(C)** One representative flow cytometry analysis of IL-4-producing T cells is shown. Normal T cells (upper panel) or SSc T cells (lower panel) were activated with CD3/CD28 magnetic beads and treated with DPI (20 μM, 1 hour). Plots represent live cells gated for CD4+ cells. Percentage of IL-4-producing T cells in three healthy controls and three SSc patients are presented (right panel). Data are means ± SD. ^*^ *P* <0.05 compared to normal untreated T cells. ^**^ *P* <0.05 compared to SSc untreated T cells. BrdU, bromodeoxyuridine; DPI, diphenylene iodonium; IFN, interferon; PMA, phorbol myristate acetate; ROS, reactive oxygen species; SSc, systemic sclerosis.

To test the biological effects of ROS on T cells, we examined proliferation and cytokine production in normal and SSc T cells. Activation of T lymphocytes with CD3/CD28 beads led to a significant increase of SSc cell proliferation compared to normal cells, expressed as ratio between SSc and normal cells (SSc/N) (Figure 7A). Treatment with NADPH oxidase inhibitor significantly decreased the proliferation rate (2 ± 0.2 vs. 1.15 ± 0.3, *P* <0.05), without influencing cell viability (Figure S4 in Additional file 5). These experiments were carried out in medium supplemented with IL-2, in order to avoid cell death after 24 hours of culturing.

Finally we measured the intracellular cytokine production in activated CD4+ lymphocytes in the presence or absence of DPI. The number of IFNγ- and IL-4-producing CD4+ T cells in SSc patients was significantly higher than in normal controls (21.1% vs. 12.5% for IFN-γ, Figure 7B; 15% vs. 7.1% for IL-4, Figure 7C; *P* <0.05). In normal cells the percentage of IFNγ- and IL-4-producing CD4+ cells was not altered by DPI treatment, while in SSc cells NADPH oxidase inhibition led to an increase of the number of cytokine-producing cells (25.1% vs. 21.1% for IFNγ, and 26.3% vs. 15 for IL-4, *P* <0.05), suggesting that IFNγ- and IL-4-production was influenced by oxidative stress (Figure 7B and C).

## Discussion

Several lines of evidence suggest that immunological mechanisms play an important role in the pathogenesis of SSc. In this respect, it is noteworthy that skin biopsies taken from SSc patients in the early stages of the disease show perivascular infiltrates consisting of activated tissue macrophages and T cells before microscopic evidence of fibrosis [5,6]. T cells in skin lesions are predominantly CD4+ cells [6], display markers of activation [9], exhibit oligoclonal expansion [35] and are predominantly type 2 helper T (Th2) [36].

Complex interactions between immune cells, fibroblasts and endothelial cells occur via cytokines, chemokines and growth factors and result in overproduction of ECM. However, a crucial role is also played by ROS as shown in previous studies [25,26] and in the present work, where we demonstrated that PBLs from SSc patients produced high levels of ROS compared to T cells from healthy controls, even in absence of deliberate stimulation (Figure 2A).

Generation of superoxide and hydrogen peroxide has been detected in T cells following stimulation of TCR [12] and, in agreement with Staal *et al*. [37], the present study showed that ROS play a mitogenic role in lymphocyte proliferation mimicking growth factors and antigenic stimulation.

In addition, another relevant finding that emerged from our study is the reduced number of CD69+ SSc T cells after exposure to DPI, suggesting that ROS are implicated in the activation of CD4+ lymphocytes (lower panels of Figure 6B and C). CD69 is a member of C-type lectin like receptor family expressed in leukocytes following stimulation [38]. Although CD69 was considered a marker of regulatory T (Treg) cells, recent studies indicated that this molecule may be involved in their immunosuppressive role. Radstake *et al*., interestingly, demonstrated that although the expression of CD69 on CD4+ SSc T cells was increased compared to controls, its expression on SSc Treg was diminished and correlated with a reduced suppressive function [39]. It remains to be established whether ROS can modulate CD69+ Treg function.

Furthermore, the present work showed that inhibition of NADPH oxidase led to a further increase of INFγ production in SSc T lymphocytes (Figure 7B). This finding was recently confirmed by Yang *et al*. [40], demonstrating that ROS scavenging in T lymphocytes from rheumatoid arthritis patients increased INFγ production. Surprisingly, our report also described an overproduction of IL-4 after ROS inhibition in SSc T lymphocytes (Figure 7C). This observation about the prevalence of cytokines, which have a distinct functional role in the pathogenesis of autoimmune diseases and fibrosis, is not new in SSc. Some studies showed the predominance of Th1 cytokines in SSc [41,42] while other reports demonstrated a Th2 profile [36]. Valentini *et al*. reported evidence of Th1 and Th2 activation in PBLs from SSc patients [43]. These conflicting data may be explained by heterogeneity within the groups studied, such as differences in the subsets of patients enrolled in the studies, duration, stage and activity of the disease, age and gender of patients and controls. Thus, further studies with well-characterized patients are needed to better clarify the link between oxidative stress and cytokine production in SSc T cells.

Interestingly we showed that, as in SSc monocytes and fibroblasts, the endogenous source of ROS is NOX2 which is overexpressed in SSc T cells both at mRNA and protein level compared to normal cells (Figure 3A and B). It is noteworthy that in SSc lymphocyte ROS generated by NOX2 induce an autocrine loop, involving activated ERK1/2 and overexpressed Ha-Ras, which maintains high levels of the oxidase and activation of the lymphocytes. This ROS-mediated loop characterizes all SSc cell lines studied so far [25,26] and thus should be considered a priority target in the development of novel anti-fibrotic therapies.

## Conclusions

Our work provided evidence that T cells isolated from SSc patients contribute to the oxidative stress of systemic sclerosis, and showed that ROS were generated by NADPH oxidase and involved in the activation of SSc T cells. These findings confirm once more the pathogenetic role of oxidative stress in SSc and indicate that NADPH oxidase may represent a potential target to be considered for the treatment of systemic sclerosis.

## Additional files

Additional file 1: Table S1. Clinical characteristics of SSc cohort. Additional file 2: Figure S1. (A) ROS production by CD4+ PBL from one SSc patient, simultaneously stained with 2 μM DCFH-DA and monoclonal antibody anti-CD4 PE for 20 minutes, was analyzed by FACS analysis. (B) ROS production by CD8+ PBL from one SSc patient, simultaneously stained with 2 μM DCFH-DA and monoclonal antibody anti-CD8 PE for 20 minutes, was analyzed by FACS analysis. Additional file 3: Figure S2. (A) Representative histogram of ROS production by PMA (100 ng/ml, 4 hours) activated normal T cells (left panel) or by SSc T cells (right panel). Untreated (grey line) and PMA activated cells (black line) were stained with 2 μM DCFH-DA and analyzed by FACS analysis. (B) Immunoblot analysis of pERK1/2 and ERK2 in cells treated as in (A). Additional file 4: Figure S3. (A) Representative histogram of ROS production by normal T cells (green line) or SSc T cells (black line) activated with CD3/CD28 magnetic beads. Cells were stained with 2 μM DCFH-DA and analyzed by FACS analysis. (B) Representative histogram of ROS production by SSc T cells activated with CD3/CD28 magnetic beads in the presence (green line) or absence of DPI (grey line). Cells were stained with 2 μM DCFH-DA and analyzed by FACS analysis. Additional file 5: Figure S4. Normal and SSc T cells activated with CD3/CD28 magnetic beads were treated with DPI (20 μM, 2 hours) and incubated with 7-AAd for 20 minutes and analyzed by FACS. One representative experiment of three is shown.

## Abbreviations

7-AAD: 7-aminoactinomycin D
BrdU: bromodeoxyuridine
conA: concanavalin A
DCFH-DA: 2′, 7′-dichlorodihydrofluorescin diacetate
DPI: diphenylene iodonium
FCS: fetal calf serum
FITC: fluorescein isothiocyanate
ERK: extracellular signal-regulated kinase
IFNα: interferon alpha
IL: interleukin
NAC: N-acetylcysteine
NOX2: NADPH oxidase 2
PB: peripheral blood
PBL: peripheral blood lymphocytes
PDGF-BB: platelet-derived growth factor beta
PerCP: peridinin chlorophyl protein
PHA: phytohemagglutinin
PMA: phorbol myristate acetate
ROS: reactive oxygen species
siRNA: small interfering RNA
SSc: systemic sclerosis
TCR: T cell receptor
TGFβ: transforming growth factor beta
Th: T helper
TNFα: tumor necrosis factor alpha
Treg: regulatory T
