# Supplementary material for: Intracellular free radical production by peripheral blood T lymphocytes from patients with systemic sclerosis: role of NADPH oxidase and ERK1/2
Source: Arthritis Res Ther. 2015 Mar 17;17(1):68. doi: 10.1186/s13075-015-0591-8 (PMC4384301; doi:10.1186/s13075-015-0591-8)
Supplement: Additional file 1: Table S1. — Clinical characteristics of SSc cohort. [file 13075_2015_591_MOESM1_ESM.pdf]

Table S1

Clinical characteristics of SSc cohort (n = 34)

M/F	6/28
mean age, years (range)	56.7 (27-84)
mean modified Rodnan skin score (range)	13.4 (1-30)
subset lSSc/dSSc	17/17
disease duration, years (range)	9.4 (1-50)
lSSc disease duration, years (range)	10.3 (1-50)
dSSc disease duration, years (range)	8.5 (1-41)

lSSc = limited SSc; dSSc = diffuse SSc
