# Supplementary figures and images for: Intracellular free radical production by peripheral blood T lymphocytes from patients with systemic sclerosis: role of NADPH oxidase and ERK1/2
Source: Arthritis Res Ther. 2015 Mar 17;17(1):68. doi: 10.1186/s13075-015-0591-8 (PMC4384301; doi:10.1186/s13075-015-0591-8)

Figure S1

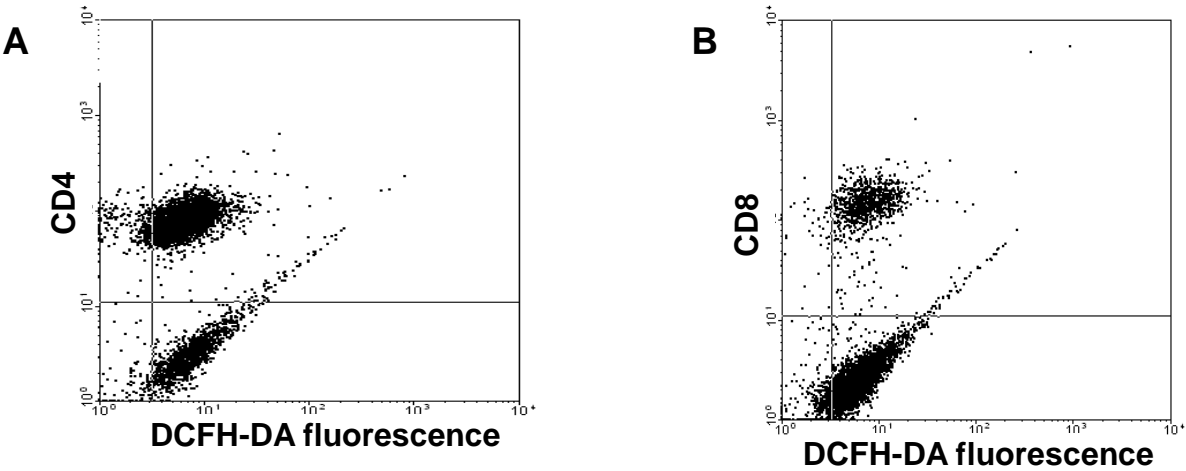

Supplement: Additional file 2: Figure S1. — (A) ROS production by CD4+ PBL from one SSc patient, simultaneously stained with 2 μM DCFH-DA and monoclonal antibody anti-CD4 PE for 20 minutes, was analyzed by FACS analysis. (B) ROS production by CD8+ PBL from one SSc patient, simultaneously stained with 2 μM DCFH-DA and monoclonal antibody anti-CD8 PE for 20 minutes, was analyzed by FACS analysis. [file 13075_2015_591_MOESM2_ESM.pdf]

Figure S2

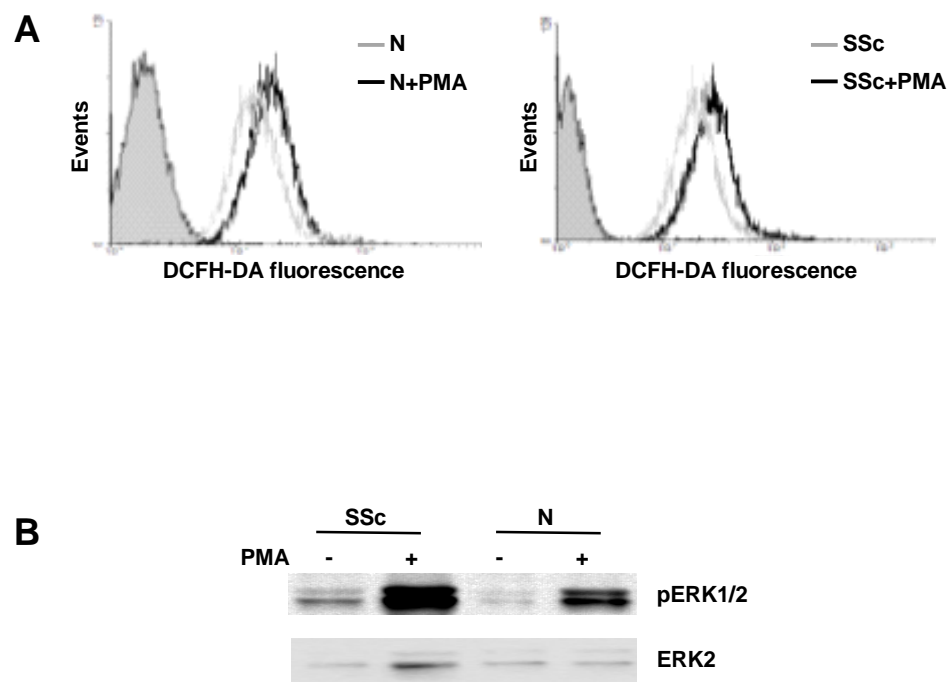

Supplement: Additional file 3: Figure S2. — (A) Representative histogram of ROS production by PMA (100 ng/ml, 4 hours) activated normal T cells (left panel) or by SSc T cells (right panel). Untreated (grey line) and PMA activated cells (black line) were stained with 2 μM DCFH-DA and analyzed by FACS analysis. (B) Immunoblot analysis of pERK1/2 and ERK2 in cells treated as in (A). [file 13075_2015_591_MOESM3_ESM.pdf]

Figure S3

A

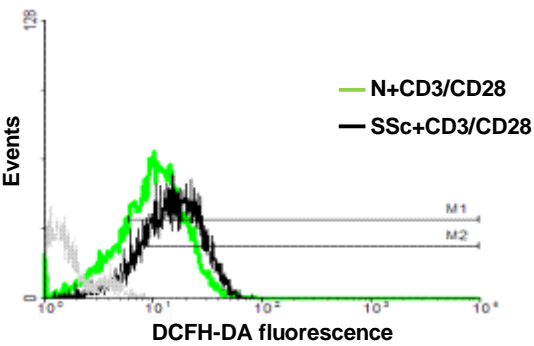

B

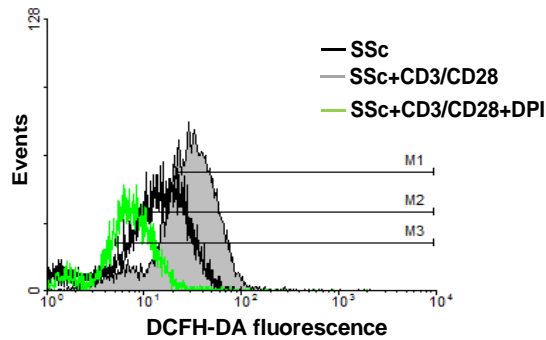

Supplement: Additional file 4: Figure S3. — (A) Representative histogram of ROS production by normal T cells (green line) or SSc T cells (black line) activated with CD3/CD28 magnetic beads. Cells were stained with 2 μM DCFH-DA and analyzed by FACS analysis. (B) Representative histogram of ROS production by SSc T cells activated with CD3/CD28 magnetic beads in the presence (green line) or absence of DPI (grey line). Cells were stained with 2 μM DCFH-DA and analyzed by FACS analysis. [file 13075_2015_591_MOESM4_ESM.pdf]

Figure S4

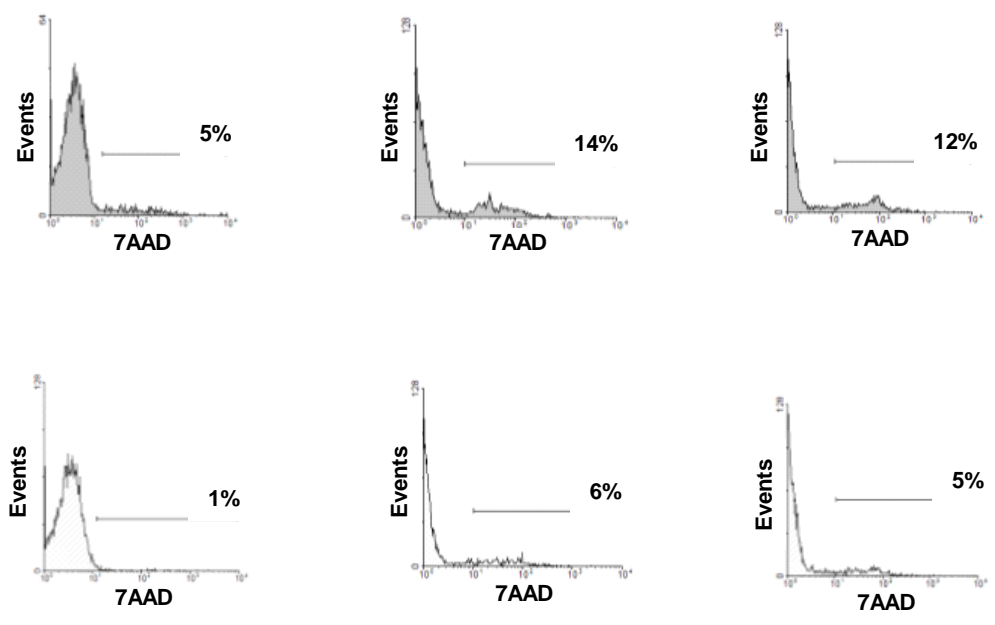

Supplement: Additional file 5: Figure S4. — Normal and SSc T cells activated with CD3/CD28 magnetic beads were treated with DPI (20 μM, 2 hours) and incubated with 7-AAd for 20 minutes and analyzed by FACS. One representative experiment of three is shown. [file 13075_2015_591_MOESM5_ESM.pdf]
